# Beneath the Skin: A Review of Current Trends and Future Prospects of Transdermal Drug Delivery Systems

**DOI:** 10.3390/pharmaceutics14061152

**Published:** 2022-05-28

**Authors:** Ahlam Zaid Alkilani, Jehad Nasereddin, Rania Hamed, Sukaina Nimrawi, Ghaid Hussein, Hadeel Abo-Zour, Ryan F. Donnelly

**Affiliations:** 1 Department of Pharmacy, Faculty of Pharmacy, Zarqa University, Zarqa 13110, Jordan; jnasereddin@zu.edu.jo (J.N.); salnimrawi@zu.edu.jo (S.N.); ghussien@zu.edu.jo (G.H.); 20189030@zu.edu.jo (H.A.-Z.); 2 Department of Pharmacy, Faculty of Pharmacy, Al-Zaytoonah University of Jordan, Amman 11733, Jordan; rania.hamed@zuj.edu.jo; 3 Medical Biology Centre, School of Pharmacy, Queen’s University Belfast, Belfast BT7 1NN, UK; r.donnelly@qub.ac.uk

**Keywords:** skin barrier, transdermal, drug delivery, permeability, microneedles, nanoparticles, niosomes, iontophoresis

## Abstract

The ideal drug delivery system has a bioavailability comparable to parenteral dosage forms but is as convenient and easy to use for the patient as oral solid dosage forms. In recent years, there has been increased interest in transdermal drug delivery (TDD) as a non-invasive delivery approach that is generally regarded as being easy to administer to more vulnerable age groups, such as paediatric and geriatric patients, while avoiding certain bioavailability concerns that arise from oral drug delivery due to poor absorbability and metabolism concerns. However, despite its many merits, TDD remains restricted to a select few drugs. The physiology of the skin poses a barrier against the feasible delivery of many drugs, limiting its applicability to only those drugs that possess physicochemical properties allowing them to be successfully delivered transdermally. Several techniques have been developed to enhance the transdermal permeability of drugs. Both chemical (e.g., thermal and mechanical) and passive (vesicle, nanoparticle, nanoemulsion, solid dispersion, and nanocrystal) techniques have been investigated to enhance the permeability of drug substances across the skin. Furthermore, hybrid approaches combining chemical penetration enhancement technologies with physical technologies are being intensively researched to improve the skin permeation of drug substances. This review aims to summarize recent trends in TDD approaches and discuss the merits and drawbacks of the various chemical, physical, and hybrid approaches currently being investigated for improving drug permeability across the skin.

## 1. Introduction

Over the last two decades, transdermal drug delivery (TDD) has received extensive interest as it has a number of advantages over conventional drug delivery systems, including its simplicity, pre-determined doses, convenience in handling, patient self-administration, and comparatively lax storage conditions. However, when it comes to drug molecules with low solubility, permeability, and degradation, the oral route of drug administration has several drawbacks [1,2,3]. The oral bioavailability of drugs varies greatly because most drugs administered orally undergo first-pass metabolism and confront many physical and biological barriers, resulting in dramatically reduced bioavailability [2,4]. Parenteral drug delivery is the most effective means for delivering drugs with a narrow therapeutic index and low bioavailability, particularly in situations where patient compliance cannot be assured (i.e., unconscious patients) [5]. However, manufacturing and administering parenteral formulations requires specific equipment, sterilization techniques, and skilled personnel [5,6]. Furthermore, the need for a low-cost, non-invasive method of vaccination, particularly in developing countries, has driven extensive study into the development of simple needle-free alternative systems, such as transdermal drug delivery (TDD). TDD is a safe and well-tolerated drug delivery approach with the potential to combine the dosing accuracy and ease of administration associated with oral dosage forms with the metabolism-free delivery of the therapeutic agent to the plasma associated with parenteral drug delivery. However, because of the low skin permeability for some drugs, its application is limited in contemporary clinical practice. The need to overcome the skin as a physiological barrier has given rise to several techniques to enhance the transdermal permeability of drugs. This review aims to summarize recent trends in TDD approaches and discuss the merits and drawbacks of the various active (physical), passive (chemical), and hybrid approaches currently being investigated for improving drug permeability across the skin.

### 1.1. An Overview of Transdermal Drug Delivery

**Figure 1:**
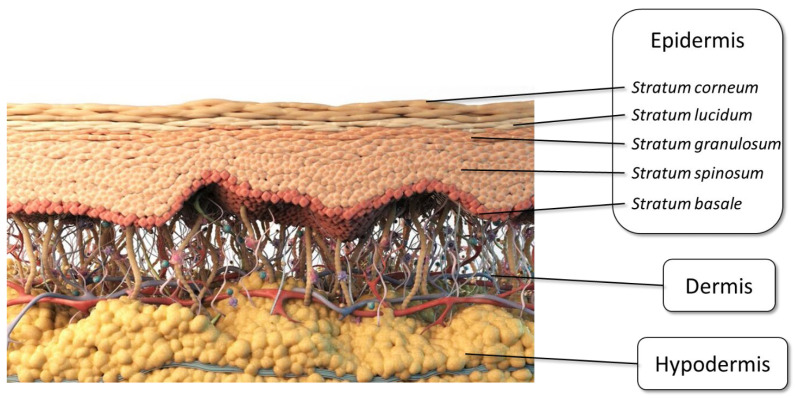
Schematic representation of the skin layers.

TDD offers many advantages over oral drug delivery, circumventing first-pass metabolism, protecting sensitive drugs from the harsh conditions of the gastrointestinal tract, and allowing for sustained release of drugs, thus maintaining a more uniform plasma concentration [7,8,9], while transdermal patches can be easily administered to children and the elderly in a safe and easy manner [10,11]. Furthermore, the use of transdermal patches has been reported to result in greater adherence in geriatric polypharmacy patients who often report swallowing difficulties and poor compliance due to the large daily pill burden [12,13]. Figure 1 shows a graphical representation of the three layers of the skin (the epidermis, the dermis, and the hypodermis).

Despite its many advantages, however, TDD is not without shortcomings. The greatest barrier to TDD is, paradoxically, the skin itself; for the drug contained within a TDD system to be absorbed into the systemic circulation, it must first penetrate the skin layers [14]. The stratified physiological structure of the skin poses the main barrier to TDD and only a select few drugs, those possessing specific physicochemical properties, are able to pass through the skin into the plasma.

Recent advances in transdermal drug delivery have given rise to several techniques and formulation strategies which can aid in overcoming the skin barrier. Said techniques are discussed in the upcoming sections.

### 1.2. Currently Approved Transdermally Delivered Drugs

The TDD market has had a considerable impact on the delivery of numerous drugs, primarily in the fields of pain management [15], hormonal applications [16], central nervous system disorders [17], cardiovascular diseases [18], and other applications, such as smoking cessation [7]. The global TDD market is anticipated to be quite large. Factors such as the prevalence of chronic diseases and technological improvements in TDD methods are leading this market forward.

In 1979, the first transdermal patch for systemic delivery was approved in the United States (Transderm Scop™; Novartis, Basel, Switzerland)—a three-day patch that delivered scopolamine to treat motion sickness [7,14]. The most recently approved patch for severe pain is buprenorphine (Butrans™; Purdue Pharma L.P, Stamford, CT, USA), approved by the FDA for the management of chronic pain that is non-responsive to other medications [19]. In addition, several over-the-counter (OTC) products are also available, including nicotine, capsaicin, and menthol patches [7].

In 2018, the first anti-histamine transdermal patch, emedastine difumarate (Allesaga™ TAPE, Hisamitsu Pharmaceutical, Tosu, Japan), indicated to treat allergic rhinitis, was approved in the Japanese market [20]. It has a dose-dependent anti-histaminic action and a long-lasting effect that lasts up to 24 h after administration [20]. In 2007, the first Parkinson’s patch containing rotigotine (Neupro™, UCB, Brussels, Belgium) was approved by the FDA—a once-daily patch that comes in four dose strengths: 2 mg, 4 mg, 6 mg, and 8 mg [17]. Rivastigmine is currently FDA-approved for administration via a transdermal patch (Exelon™, Novartis) for the treatment of Alzheimer’s disease [13]; the patch overcomes gastrointestinal (GI) adverse effects associated with oral rivastigmine [21]. Ortho Evra™ is an FDA-approved transdermal ethinyl estradiol and norelgestromin contraceptive patch. The patch is applied once-a-week for three weeks (21 days), with one patch-free week included in the cycle [7,16]. Apleek™ is a transdermal contraceptive patch containing 550 micrograms of ethinylestradiol and 2.10 mg of gestodene as active ingredients; it is applied once a week for three weeks, followed by a seven-day patch-free period [22].

## 2. Techniques for Enhancement of Skin Permeabilisation

**Figure 2:**
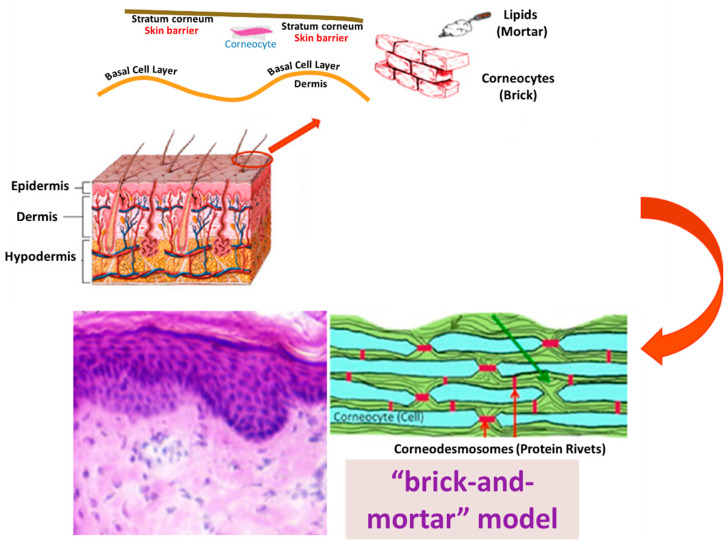
Structure of the SC. Adapted with permission from [24,29].

**Figure 3:**
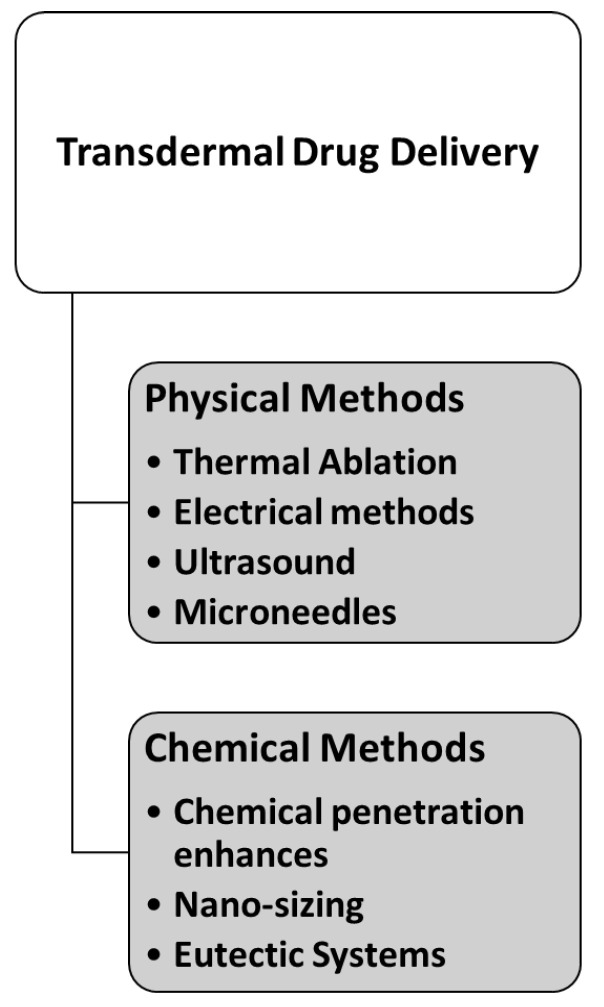
Approaches for enhancing drug transport across the skin.

Despite its many merits, TDD is restricted to a small number of drugs with specific physicochemical properties. A drug candidate for transdermal delivery should ideally have a molecular weight of less than 500 Da with a moderate lipophilicity (log P range 1–3) to pass freely through the skin, whereas hydrophilic and macromolecular drugs, such as peptides, are frequently hampered by the barrier of the stratum corneum (SC) [8,9,14,23]. The SC is a 5–20 µm-thick skin layer that acts as a main barrier [8,9,14,23] against the outside environment. The SC is dense and impermeable to drug molecules due to the 10–15 layers of corneocytes, lipid matrix, corneodesmosomes, and tight junctions comprising its structure (Figure 2) [24,25,26]. As a result, the most challenging aspect of TDD is to overcome the SC barrier, deliver the drug to the skin, and allow the drug to diffuse to reach the blood vessels in the dermis. The issue is that only a small number of drugs can get through the skin, so the application of TDD is limited in clinical practice. To circumvent this constraint, it is advised that new and novel TDD approaches for skin penetration improvement be developed to overcome these challenges. Thus, some prospective strategies, including both chemical and physical methods, have been investigated in order to overcome the SC barrier [8,9,14,24,27]. The methods utilized to modify the barrier properties of the SC can be classified as chemical and physical methods, as summarized in Figure 3 [14,28].

Prausnitz and Langer described three generations of TDD systems in a paper published in 2008 [8]. The first generation of TDD systems consisted of passive dosage forms (i.e., transdermal patches) which were used for the delivery of small, lipophilic, potent drugs and few attempts were made to enhance their skin permeability. The second generation of TDD systems included dosage forms which utilized chemical enhancers and iontophoresis [8]. The third generation of TDD systems is characterized by systems that rely on SC disruption, rather than penetration enhancement, for permeation enhancement. Third-generation TDD systems include electroporation, microneedles (MNs), and thermal ablation techniques, all of which have been documented to allow the permeation of large macromolecules across the skin, including proteins and vaccines, and they have demonstrated the capacity to deliver a much broader range of medications. It is worth noting that, as shown in Figure 3, the majority of penetration enhancement methods commonly associated with second-generation TDD systems tend to be chemical methods, while the SC-disruptive methods utilized in third-generation TDD systems tend to be physical methods. Upcoming sections discuss newer approaches in which TDD systems utilizing a combination of chemical and physical approaches for penetration enhancement are being investigated. These so-called hybrid systems can be thought of as the “fourth generation” of TDD systems and are discussed in detail in the upcoming sections.

### 2.1. Chemical Methods for Transdermal Drug Delivery

Chemical methods are used to improve the permeability of drugs across the SC via affecting drug and vehicle interaction, as well as formulation optimization [10,14]. Chemical methods are the most commonly explored approach to transdermal drug permeation enhancement since they are relatively affordable and simple to produce, offer design flexibility, and allow patients to self-administer their drugs [10,30,31]. In addition, chemical methods can be combined with drugs to produce creams or gels, as well as skin patches, that can be applied anywhere on the body for short- and long-term systemic administration [30]. However, the rate of drug diffusion using this approach is primarily determined by the molecular weight as well as the concentration gradient of the drug, making it difficult, if not impossible, to distribute macromolecules through the skin. In fact, the need for a sufficient concentration gradient across the skin membrane leads to a rate-limiting step; for a drug to diffuse across the skin, it must first be available on the skin surface in sufficiently high concentrations to create a concentration gradient that will act as the driving force for diffusion across the SC. The time needed for sufficient amounts of the drug to be released from the transdermal dosage form (i.e., a transdermal patch) and accumulate on the skin surface in sufficiently high concentrations to facilitate transdermal penetration is called the lag time. The most important aspects of the pharmacokinetics of transdermal patches are lag time and bioavailability [32]. The lag time in drug release may be the most notable drawback of the chemical approach. Dosage forms which exhibit a lag time are unsuitable for use when an early onset of action is desired [14,33]. In addition, the bioavailability of transdermal patches is sometimes low when compared with parenteral dosage forms. For example, the transdermal bioavailability of rotigotine is about 37%. In another study of 51 Alzheimer’s disease patients, the bioavailability of a rivastigmine transdermal patch was reported to be approximately 50% of the entire loading dosage [34].

#### 2.1.1. Chemical Penetration Enhancers (CPEs)

The most commonly explored chemical method used for modifying the barrier properties of the SC is the use of chemical penetration enhancers (CPEs) [35,36]. CPEs are substances that have been examined for their capacity to boost drug molecule transport across the skin [8,30,35]. They achieve their action through a variety of mechanisms that are dependent on the chemical composition of CPEs, such as disrupting the organized lipid bilayer, interacting with cell membrane proteins, interacting with intercellular proteins, disruption of intercellular lipids, enhancing hydration in the SC, and affecting the partition coefficients of drugs [14,37,38,39,40,41].

More than 300 CPEs have been used in different TDDs to facilitate the passage of drugs through the SC [30]. The optimum enhancer should be nontoxic and bio-compatible and its activity and duration of action should be predictable and consistent at the same time. CPEs should promote the transport of drugs into the body while preventing the loss of endogenous materials (unidirectional flow) [38,39,42,43,44].

The most frequently used CPEs are alcohols, sulphoxides, azone, pyrrolidones, essential oils, terpenes, fatty acids, and urea (Table 1) [14,44]. Combinations of CPEs provide a number of ways to get beyond the limits of single chemical enhancers. Therefore, the use of combinations of CPEs has been investigated and approved to enhance permeability and reduce irritation [8,37,38,45]. Aside from their positive effects on the rates of drug transport, CPE combinations have been reported to improve the potency and safety of transdermal dosage forms. Karande et al. [46] demonstrated the feasibility of systemic delivery of macromolecules from a transdermal patch employing a combination of penetration enhancers (sodium lauryl sulfate and phenyl piperazine). Additionally, Bozdaganyan et al. [47] reported that using two CPEs (linoleic acid and ethanol) on an SC model enhanced the delivery of lidocaine in a synergistic way [47]. Moreover, the effect of a penetration enhancer on the permeation of atenolol through excised rat skin was investigated by Cho et al. [48]. In comparison with glycols, fatty acids, and non-ionic surfactants, polyoxyethylene 2-oleyl ether was the most effective enhancer. It showed an increase in flux, which was most likely related to an enhancement of skin permeation [48]. Although CPEs tend to give 10-fold maximum increases in permeation for some drugs, they have a major constraint when it comes to hydrophilic macromolecules such as insulin and other proteins. Indeed, none of these treatments has yet been commercialized as a transdermal patch. The main drawback of CPEs is poor safety and efficiency [14,30,38,49]. Skin irritation, rather than a dangerous lack of safety, tend to be the biggest issues with penetration enhancers [50].

#### 2.1.2. Vesicles

**Figure 4:**
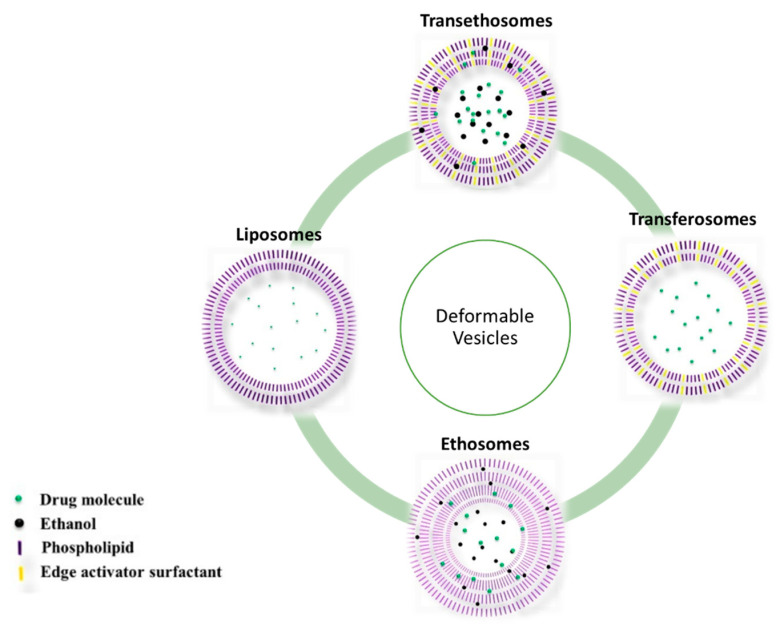
Schematic representation of the different types of vesicular carriers, with defined layers and compositions. Adapted with permission from [88].

One of the most promising chemical methods developed to penetrate the SC is the use of vesicular systems, particularly nanosized systems. They can increase the bioavailability of encapsulated drugs while also enabling therapeutic activity in a controlled manner [73,74,75,76]. These systems were found to be capable of increasing drug residence time in the epidermis while also modulating systemic absorption [76,77]. Vesicles are highly organized assemblies made up of one or more bilayers as a result of the self-assembly of amphiphilic building blocks in the presence of water [78]. They are systems that can improve the bioavailability of drugs and reduce toxicity by targeting specific sites [79]. Furthermore, problems of drug instability, insolubility, and rapid degradation can often be mitigated by incorporating drugs into vesicles [76,80]. The characteristics of vesicles, rather than the physicochemical properties of drug molecules, control the clearance and tissue distribution profile of a drug when delivered by such a delivery system [81]. Based on the physicochemical characteristics of the drug, it can be encapsulated in the internal cavity or be included in the bilayer [28]. Hence, vesicles can load hydrophilic, lipophilic, and amphiphilic drugs to achieve transdermal delivery [10]. Vesicles can be classified as liposomes, transferosomes, and niosomes, depending on their constituent molecules, as shown in Figure 4 [10]. Lipid-based vesicles have been widely used as vehicles to encapsulate and deliver drugs since Alec D. Bangham’s discovery of liposomes in 1965 [75].

Liposomes are vesicular systems with an aqueous internal environment made up of phospholipids and fatty acids that are essentially biocompatible and biodegradable due to their natural abundance in cell membranes [82,83]. They form lamellar sheets when dispersed in aqueous media by aligning themselves in such a way that the polar head groups face outwards towards the aqueous region while fatty acid groups face each other, forming spherical, vesicle-like structures [84]. Deformable liposomes consist of phospholipids, surfactants, and an inner aqueous compartment enclosed within a lipid bilayer capable of encapsulating hydrophilic and lipophilic drugs [85]. Surfactants play the role of the edge activator, destabilizing lipid bilayers and increasing vesicle deformability [86,87]. Flexible, elastic, ultra-deformable, and ultra-flexible liposomes are all names for deformable liposomes [83]. When compared with conventional liposomes, results have shown deformable liposomes to have superior therapeutic impacts, as they were found to penetrate deeper into skin layers [75,76,86].

The first generation of elastic liposomes is comprised of transfersomes [83]. Transfersomes, also known as ultra-flexible liposomes, are elastic vesicles with a backbone made of phospholipids and an edge activator [76]. The edge activator, which weakens the lipoidal bilayer of the vesicles and increases their flexibility and deformability, is primarily responsible for their elasticity [83]. Transfersomes are generally prepared using the thin-film hydration method [89]. The deformability of transfersomes and the osmotic gradient across the application site play a significant role in their enhanced penetration into the skin [90,91]. Duangjit et al. [92] reported that meloxicam-loaded transfersomes may be used as a TDD system because they showed much higher meloxicam skin permeation than liposomes. Due to their ultra-deformability, transfersomes are widely used by researchers as transdermal delivery carriers for anti-cancer agents [89], non-steroidal anti-inflammatory drugs [93], anesthetics [94], insulin [95], and corticosteroids [96]. Although transfersomal delivery systems have various advantages, they are chemically unstable, phospholipid purity is another key factor that has to be considered, and they are costly [90].

The development of surfactant vesicles (niosomes) as a means of improving TDD has attracted the interest of scientists working in the field of drug delivery systems in recent decades. Niosomes are self-assembled vesicular nanocarriers made by hydrating synthetic surfactants with cholesterol or other amphiphilic compounds in appropriate quantities [74,97]. Niosomes, like liposomes, can be unilamellar or multilamellar and can carry both hydrophilic and lipophilic drugs, while also delivering them to targets [74,98,99,100]. Furthermore, niosomes are considerably more stable during the formulation and storage procedures than liposomes. [74,98,99]. Surface modification or component optimization can be used to attain the desired pharmacokinetic qualities, since they can control drug release, resulting in the lower toxicity, better targeting, and improved bioavailability of encapsulated drugs [101,102]. This innovative delivery system is also simple to create and scale up at a cheap cost of manufacture [101].

The thermodynamic and physicochemical parameters, such as the hydrophilic–lipophilic balance (HLB), are the most important factors in the preparation of niosomes. However, several other factors are important for vesicle formation and must be considered, including the hydration medium, the physicochemical properties of encapsulated drugs, lipid-chain length, cholesterol content, and additives. A variety of methods for preparing niosomes have been described in the literature, including thin-film hydration, ether injection, sonication, and reverse phase evaporation; however, the properties of niosomes vary greatly depending on the method utilized for manufacture [74,101,102].

El-Ridy et al. [103] explored the use of lornoxicam niosomal gels for transdermal delivery to increase permeability and anti-inflammatory efficacy using the thin-film method. They found that the percentage of edema inhibition achieved using lornoxicam niosomes was substantially higher than that achieved using free lornoxicam, indicating that lornoxicam niosomes have increased anti-inflammatory efficacy [103]. Similarly, lopinavir niosomes were made via the thin-film hydration process and optimized using Span 40 and cholesterol [104]. An in vivo bioavailability study in male Wistar rats revealed that lopinavir was absorbed considerably better via a transdermally administered niosomal gel than via oral solution [104]. Although there are some reports of successful commercialization—cosmetic manufacturers Lancôme and L’Oréal have developed niosomal formulations of anti-aging active compounds and there are currently clinical trials investigating urea and griseofulvin niosomal gels—there remain very few reports on the successful commercialization of niosome-based products, particularly formulations of pharmaceutical products for chronic health conditions.

It should be pointed out that, despite their reported effects in enhancing transdermal penetration, evidence suggests that they have little to no effect on the plasma availability of transdermally delivered dosage forms. Furthermore, the exact mechanism by which they enhance drug penetration across the SC is still a point of contention; some reports suggest that transfersomes remain intact and are taken up by the skin and into the bloodstream [105], while other reports disagree with this theory [106,107], citing their negligible effect on plasma availability as evidence to the contrary.

#### 2.1.3. Nanoemulsions (NEs)

Nanoemulsions (NEs) are thermodynamically stable, isotropically clear dispersions of oil and water that are stabilized by an interfacial film of surfactants and co-surfactants with droplet size ranges between 10 and 100 nm [108,109]. NEs are also called miniemulsions, submicron emulsions, and ultrafine emulsions [108]. Although NEs have almost the same droplet composition and appearance as microemulsions, they differ notably in terms of structural aspects and long-term thermodynamic stability [10]. For instance, NEs are thermodynamically unstable due to the free energy of NEs (droplets in water) being higher than those of the separate phases (oil and water), whereas microemulsions are thermodynamically stable, owing to the fact that the free energy of a microemulsion is lower than those of the separate phases [110,111]. Due to the difference in the thermodynamic stabilities of colloidal dispersions, the formation of NEs requires an external energy supply to rupture larger droplets into smaller ones, whereas microemulsions are formed by simply mixing components at a particular temperature without the use of any external energy or device [111,112,113].

NEs are prepared using several methods classified into high-energy methods and low-energy methods [110]. The high-energy methods include microfluidization [114], high-pressure homogenization [115], and ultrasonication [116], whereas the low-energy methods include emulsion inversion point (EIP) [117], phase inversion temperature (PIT) [118], and spontaneous emulsification [119]. The selection of any method depends on the drug itself and the dosage form. In addition, surfactants must be chosen carefully to obtain an ultra-low interfacial tension (<10^−3^ mN/m) and to obtain droplet sizes with stable emulsion systems [120].

**Figure 5:**
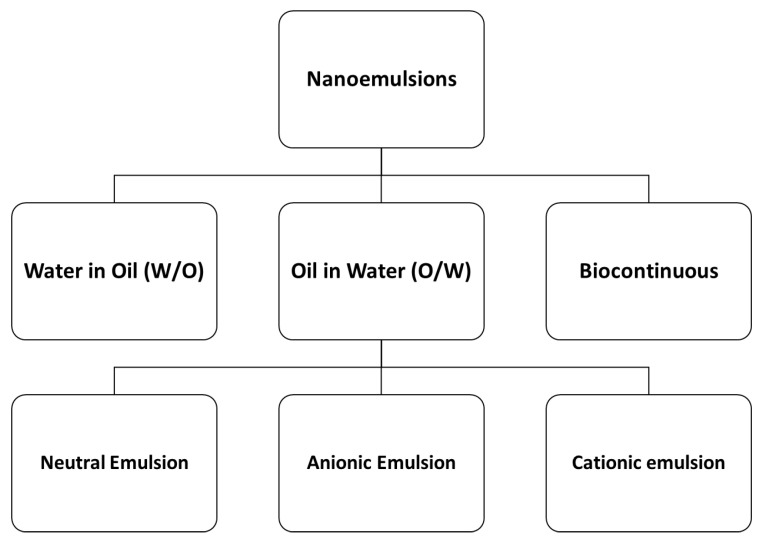
Types of NEs.

Based on the composition of oil and water portions, NEs are classified into three types: (1) oil in water (O/W) NEs, where the oil droplets are dispersed in a continuous aqueous phase; (2) water in oil (W/O) Nes, where the water droplets are dispersed in a continuous oil phase; and (3) bi-continuous NEs, where the microdomains of oil and water are inter-dispersed within the system [121]. Based on the type of surfactants used in O/W NEs, these NEs are further classified into three types [122]: (1) neutral O/W Nes, where neutral surfactants, such as Tweens and Spans, are most commonly used [123,124]; (2) cationic O/W NEs, where cationic surfactants, such as quaternary ammonium compounds and 1,2-Dioleoyl-3-trimethylammonium propane (DOTAP), are used in ophthalmic [125] and gene delivery [126]; and (3) anionic O/W NEs, which are prepared with the anionic surfactant sodium dodecyl benzene sulphonate (SDBS) using the PIT method [127] (Figure 5).

NEs, compared to other TDDs, are far more efficient as drug delivery systems. This is because NEs ensure close contact with the skin due to their excellent wettability, small droplet sizes, large specific surface areas, and low interfacial tension [128,129]. In addition, NEs offer many other benefits, such as high solubilization capacity and physical stability, long shelf-life, ease of preparation, production with less energy input, improved bioavailability, greater absorption (due to smaller droplet size and thus greater surface area), incorporation of non-irritant and non-toxic components, and the protection of drugs from degradation [108,130,131].

NEs have been used as nanocarriers to enhance the transdermal delivery of a wide range of hydrophilic or hydrophobic active compounds, such as nonsteroidal anti-inflammatory drugs (NSAIDs), anticancer drugs, and antioxidants, among others [132,133]. Drug-loaded NEs can be either applied directly onto the skin or incorporated within secondary delivery systems, such as gels, films, or patches, to further enhance the physicochemical properties of the drugs, improving penetration and sustaining release. Table 2 summarizes the drugs loaded into NEs, the types of NEs, the method of NE preparation, drug applications, and the transdermal delivery systems.

#### 2.1.4. Nanoparticles

Nanotechnology is applied in medicine to deliver nanoscale particles that are able to penetrate cell membranes [164,165]. Nanoparticles (NPs) are solid colloidal particles ranging in size from 10 to 100 nm [166]. The utilization of NPs in the pharmaceutical industry has been increasing over time. The small size of NPs makes them capable of moving through various biological barriers to bring drugs to target sites, resulting in greater bioavailability and therapeutic efficacy [167]. However, in the case of skin, only a very small number of NPs can cross the skin barrier.

**Figure 6:**
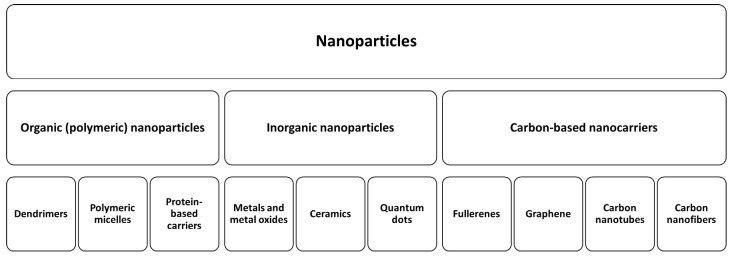
Classification of nanoparticles.

As discussed in a previous section, one of the main disadvantages of CPEs is their likelihood of inducing irritation, damage, and reductions in skin barrier function [168,169]. As a result, the use of NPs as TDD carriers is gaining in popularity; NPs can carry or deliver a variety of therapeutic and diagnostic agents, such as small and large molecules, proteins, and nucleic acids, and then release them in a controlled manner [170]. Moreover, NPs can improve the solubility and stability of drugs, providing an opportunity to evaluate drug candidates that were ignored previously because of poor solubility and variable bioavailability [170]. Lastly, NPs can be delivered through various routes of administration that may achieve site-specific drug delivery [170]. NPs have been studied for application in the transdermal delivery of vaccines [171], anti-hypertensives [172], anti-cancer agents [173], and many other drugs [174]. NPs are generally classified into three types: organic (polymeric), inorganic, and carbon-based NPs [166] (Figure 6).

Organic (polymeric) NPs are fabricated by the polymerization and crosslinking of biodegradable polymers [175,176]. Such polymeric nanoparticles are often fabricated by inducing the “self-assembly” of the polymer—a process by which the polymer is forced to assume a coiled, particulate form as opposed to an uncoiled chain conformation. The most commonly used polymer is chitosan [177]. However, other natural and semisynthetic polymers have been investigated for the formulation of nanoparticles, including Poly(lactide-co-glycolide) (PLGA) [178], poly lactic acid (PLA) [179], and cellulose nanoparticles [180], among others [181]. Polymeric NPs are characterized by high mechanical strength and cannot pass through pores with dimensions smaller or equal to their size. However, because these NPs are difficult to break down, drugs can be held for a long time before being released from the NPs and diffusing into the deeper layers of the skin [10]. Several studies have reported the use of NPs as TDD systems. For instance, Hoang Nhan Ho et al. [182] revealed that a prolonged release profile and good penetration of itraconazole through mouse skin were achieved using polymeric NPs with colloidal sizes in the range of 200 nm. In addition, they found that the incorporation of itraconazole-loaded NPs into a gel formulation for TDD has the potential to improve antifungal activity with respect to the conventional gel. Woo Yeup Jeong et al. [183] used a solvent evaporation approach to prepare minoxidil polymeric NPs. Cell viability, cellular uptake, and skin permeation tests revealed that polymeric NPs delivered adequate amounts of minoxidil to cells without causing significant cytotoxicity.

Inorganic NPs are currently the subject of extensive attention for their potential use in TDD since they have a tunable particle size and offer superior chemical and mechanical stability in relation to polymeric NPs, as well as easier surface functionalization [184]. As a result, one of the fastest expanding disciplines in nanotechnology is the development of innovative transdermal systems based on inorganic NPs [185]. Some inorganic NPs possess a unique property, such as antimicrobial function, anticancer activity, or light-scattering effects for photoprotection [186,187]. These NPs include silver nanoparticles (AgNPs), titanium dioxide nanoparticles (TiO2NPs), zinc oxide nanoparticles (ZnONPs), and gold nanoparticles (AuNPs) [188,189]. A number of studies have focused on the use of inorganic NPs as promising nanocarriers in TDD in recent decades [190,191]. Colchicine transdermal administration was found to be difficult because of its high water solubility and concomitantly low skin permeability. Therefore, Amina et al. [192] used inorganic NPs of mesoporous silica as colchicine encapsulators and incorporated them in a hydrogel transdermal patch. The therapeutic examination of colchicine-formulated transdermal patches in a mono-iodoacetate (MIA)-induced rat osteoarthritis model revealed increased locomotor activity, glutathione blood levels, and a significant decrease in malondialdehyde, nitric oxide, and COX-2 levels. The observed results demonstrated that the developed colchicine NP-loaded hydrogel patches showed substantial promise of providing an effective, safe, and patient-friendly formulation for osteoarthritis therapy [192].

Carbon-based nanomaterials (CBNs) have attracted a great deal of attention in TDD research because of their unique structural dimensions and physicochemical features [193]. Strasinger et al. [194] reported the use of various electrical biases to test the transdermal delivery of clonidine mediated by a carbon nanotube–epoxy nanocomposite membrane. According to therapeutic feasibility studies, the carbon nanotube membrane acted as the rate-limiting step in clonidine diffusion, and the lag and transition periods were adequate for clonidine therapy. This study effectively demonstrated the use of switchable carbon nanotube membranes to deliver therapeutic flux values of clonidine transdermally for the treatment of opioid withdrawal symptoms [194].

#### 2.1.5. Nanocrystals

Particle size engineering is perhaps one of the oldest and most studied methods of enhancing drug bioavailability. The increase in available surface area brought about by the reduction of particle size has long been documented as enhancing drug dissolution rates [195,196,197] and the micronization of pharmaceutical powders continues to be one of the most common methods used to obtain a more favorable drug release profile [198]. The use of nanocrystals as a formulation strategy can be thought of as the latest iteration of particle size engineering as a means to improve drug bioavailability [199].

A nanocrystal is generically regarded as a pure drug crystal whose size falls in the sub-micron (<1000 nm) range [200]. Employing nanocrystallization is a lucrative drug delivery strategy due to the advantages offered by the relevant systems. Nanocrystals can be administered through various routes of administration [201,202,203,204,205] and they offer much improved dissolution rates over traditional delivery systems [203,204,206]. Furthermore, since typical nanocrystal formulations generically consist of pure drug crystals, they are less likely to raise biocompatibility and toxicological concerns that are typically associated with formulations that have more complex constituents [207]. Nanocrystals have gained considerable interest for topical and transdermal drug delivery. They often possess higher loading efficiencies compared to other nanotechnology-based delivery systems, as they contain mostly pure drug crystals while having a comparatively simpler formulation (nanocrystal formulations typically consist of pure drug crystals coupled with small amounts of stabilizers, such as surfactants or polymers) [208,209].

Compared to other TDDs, nanocrystals employ chemical methods to enhance drug penetration through the skin [210]. The increased surface area brought about by the nanonization of drug crystals yields a significant increase in the apparent solubility (Cs) levels of drugs [211]. When applied to the skin, their increased Cs levels result in rapid dissolution on the skin, creating a supersaturated solution atop the skin and a concentration gradient across the skin membrane. This concentration gradient serves as the driving force for passive diffusion across the skin, where the dissolved active ingredient in the aforementioned supersaturated solution diffuses through the skin (either intracellularly or transcellularly) and is rapidly replenished from the formulation with yet more drug [200,210,211]. Furthermore, nanocrystals have better biological adhesion, which allows them to stay on the skin surface for longer periods of time, maintain a high concentration gradient for longer periods of time, and facilitate drug molecule absorption into the skin. Undissolved nanocrystals can aggregate in hair follicles to produce a drug reservoir in addition to intracellular and intercellular pathways [212]. Not only nanocrystals but all other types of nanoparticles can accumulate in hair follicle canals and form reservoirs.

Due to the aforementioned advantages, there is significant research interest in the use of nanocrystals as vehicles for TDD. Eckert et al. [213] reported a transdermal film formulation consisting of curcumin nanocrystals that showed a strong correlation between the number of nanocrystals present in the film and the penetration ability of the curcumin. Wadhawan et al. [192] reported the use of nanocrystals to enhance the intra-dermal penetration of acyclovir, demonstrating an increase in drug penetration 6.4 times that of the marketed product [214]. Khan et al. [215] demonstrated an improvement in the anti-inflammatory effect of capsaicin over the marketed product when capsaicin was incorporated as a nanocrystalline formulation. The various research efforts into the use of nanocrystals as means of drug delivery serve to highlight their flexibility as drug delivery systems. The aforementioned research by Eckert et al. and Wadhawan et al. incorporated nanocrystal formulations into adhesive films [213] and semi-solid formulations [214], respectively. Additionally, Tekko et al. [216] reported a sustained-release MN formulation incorporating methotrexate nanocrystals. Moreover, Avasatthi et al. [217] incorporated methotrexate nanocrystals into a gel formulation for TDD. Table 3 presents an overview of some research efforts into nanocrystal formulations, highlighting the dosage forms of choice for said formulations.

#### 2.1.6. Solid Dispersions

Solid dispersions are yet another well-studied means of enhancing the bioavailability of poorly water-soluble drugs [197,225]. The term “solid dispersion” has been used to describe a multitude of different systems, including eutectic mixtures, glass solutions, glass suspensions, and inverted solid dispersions [226]. However, the term has since come to be more clearly defined and to refer to a two-component system that consists of a drug that is molecularly dispersed in a continuous polymeric phase [227]—a system that has come to be known as an amorphous solid dispersion (ASD). ASDs have been thoroughly investigated as means to improve the bioavailability of Biopharmaceutical Classification System (BCS) Class II drugs [198,227,228,229,230,231,232]. Perhaps the key advantage of ASDs over other dissolution enhancement strategies is processing flexibility; ASDs can be prepared by a number of different methods, most notably spray drying, freeze drying, and hot-melt extrusion [207,227,230,231,233].

The use of solid dispersions has been documented as a means to enhance transdermal drug penetration [200,234,235,236,237]. The mechanism of permeation enhancement employed by ASDs is functionally identical to that employed by nanocrystals [229,235,236]. The much-improved dissolution rate of ASDs creates a supersaturated solution atop the skin, which results in a concentration gradient that is the driving force of drug penetration. However, unlike nanocrystals, solid dispersions offer one notable advantage in that, due to the incorporation of the drug within the polymeric matrix, there is no need to further process the formulation, as the polymeric matrix can serve both as a stabilizer for the amorphous drug and as a transdermal patch/dosage form. Marreto et al. [238] reported melt-extruded transdermal patches of carvedilol ASDs incorporated in Soluplus™. In addition, Azizoglu et al. [239] reported melt-extrusion/3D printing of transdermal patches containing montelukast sodium ASDs. Similarly, Chaudhari et al. [240] reported the formulation of quercetin ASDs prepared by the melt-extrusion/3D printing of transdermal patches. Solvent-casting ASDs to prepare transdermal patches has also been reported [241,242,243].

### 2.2. Physical Methods for Transdermal Drug Delivery

Due to the barrier properties of the SC, conventional approaches relying on passive diffusion of drug molecules into the skin are unable to deliver macromolecules, such as peptides, proteins, DNA, and vaccines. Over a period of almost 35 years, the focus has been not just on overcoming the skin’s barrier property but also on the safety, accuracy, and patient compliance aspects of conventional methods [244]. The previous limitations can be solved by using physical techniques to change the SC barrier function [10,244]. To increase drug transport via the skin, physical techniques utilize external energy as a driving force or physically disrupt the SC [14,244]. Many drugs, including lipophilic and hydrophilic molecules, vaccines, and macromolecules, can be delivered. In comparison with chemical approaches, this method provides more control over drug delivery patterns, resulting in a shorter lag time [14,33,244]. Furthermore, the devices and their application parameters can be tailored to the skin characteristics of each patient. Many techniques, such as iontophoresis, high-velocity jets, and MNs, have been successfully used under the physical approach [10].

#### 2.2.1. Electrical Techniques

The two major techniques of electrically-facilitated TDD include electroporation and iontophoresis. Applying high intensities of electric pulses on skin cells leads to the formation of aqueous pores and other structural rearrangements in the lipid membranes of the SC, allowing the diffusion of drugs across the skin. This biophysical phenomenon is called electroporation or electro-permeabilization [245,246]. The electric pulses applied for milliseconds allow the diffusion of drugs through long-lived electropores for up to several hours [246]. Electrophoresis has been employed in the delivery of charged moderate and large molecules across the skin [247]. Pulse parameters, such as duration, number, and shape, in addition to field strength, are all factors that control both skin permeabilization and drug transport across the skin [248]. Although electroporation is considered safe for the skin, the complexity of the device designed for electroporation limits its use in TDD in humans [246]. Therefore, it has a minor application in TDD compared to iontophoresis.

Iontophoresis is a technique which involves the application of an electric current (0.1–1.0 mA/cm^2^) to introduce ionized and neutral drugs into the skin [14,249,250]. There are two principal mechanisms of enhancing drug transport across the skin into systemic circulation via iontophoresis: electromigration and electroosmosis [251].

Electromigration is the ordered movement of ionized drug molecules in the presence of an electric current; charged drugs are forced across the skin by electronic repulsion of similar charges [249]. Positively charged and negatively charged electrodes can diffuse cationic and anionic drugs through skin, respectively [252]. Electroosmosis is the movement of fluid containing hydrated ions in the presence of an electric current [252]. Skin has a slight negative charge at physiological pH. Thus, electroosmotic flow occurs from the anode (positive electrode) to the cathode (negative electrode), so the force of introducing cations from the positive electrode includes the repulsive force from the electric current in addition to the force generated by electroosmosis [249,253,254]. Electroosmotic flow plays a dominant role during the passage of neutral particles thought the skin [250].

Various parameters affect the iontophoresis technique, including the pH of the donor solution, electrode type, ionic strength, buffer concentration, current strength, and the type of current employed [14,249,255,256,257]. In addition, the particle size of the drug is one of the most important factors that determines the feasibility of successful iontophoresis, smaller and more hydrophilic ions being fluxed faster through the skin than larger ions [258,259]. In addition, there is a linear relationship between the electrical current and drug flux across the skin [14]. Moreover, the current limitation to 1 mA is one of the main drawbacks in iontophoresis, where a low electrical current is necessary to facilitate patient comfort and avoid the risk of nonspecific vasodilatation reactions, which is elevated by increasing the current [252]. Furthermore, local skin irritation or burns might happen if the device is applied for more than 3 min or if the electrical current is above 0.5 mA/cm^2^ [260,261,262]. In addition, polarization effects on the skin can be induced by using continuous direct current; thus, a pulsed current has been used instead [14]. Iontophoresis has minor effects on skin structure over short treatment periods due to the low-voltage nature of the applied electric current as compared to electroporation [263].

Proteins and peptides are considered ideal candidates for iontophoresis as they are usually charged at physiological pH or can have their charges altered by altering pH. Additionally, the molecular weight and mobility of peptides are reported to affect drug permeation via iontophoresis [264]. Furthermore, iontophoresis has been used in pediatric anesthesia, e.g., lidocaine iontophoresis [265]. Iontophoresis has also been used in diagnostic applications, e.g., in the diagnosis of cystic fibrosis using pilocarpine iontophoresis [266] and for monitoring blood glucose levels by reverse iontophoresis [267]. There are some examples of commercially available iontophoretic delivery systems, e.g., Lidosite™ for lidocaine [252], Ionsys™ [268] for fentanyl, Zecuity™ for sumatriptan [269], and a facial spa device by NuFace™ for facial toning without the use of chemical agents [270]. Although iontophoresis does appear to offer numerous advantages, there are still concerns about its safety which are intimately linked to the efficiency of iontophoresis devices. Furthermore, iontophoresis devices are generally expensive and quite complex, rendering them a less favourable approach from the perspective of patient compliance. Moreover, the corrosion of the metal components of electrodes upon storage remains an issue [271]. The aforementioned disadvantages have bottlenecked the commercial adaptation of iontophoresis as a transdermal delivery system.

#### 2.2.2. High Pressure-Based Devices

High pressure-based or velocity-based delivery devices such as jet injections have recently joined the battery of transdermal delivery enhancement techniques, the need to deliver high amounts of therapeutics at higher speeds having driven the development of such devices [14,39]. An attractive alternative to needle-based injection, the needle-free jet injector is a device that generates high-speed (120–200 m/s) jets of either powder or liquid jet injections that puncture the skin and deliver drugs using a power source such as compressed gas or a spring [14,272]. Although jet injections rupture the epidermal layer, which is reversible in nature [273], this technique is known to be painless and non-invasive [14]. Additionally, needle-free jet injectors offer other advantages for transdermal delivery, such as greater patient compliance, especially in chronic disease cases, and the minimization of infections and disease transmissions that result from improper reuse of needles [274]. As the delivery of drugs via jet injection is not dependent on their diffusion rates, this method overcomes the limits of existing drug delivery technologies, such as iontophoresis and electroporation [274].

**Figure 7:**
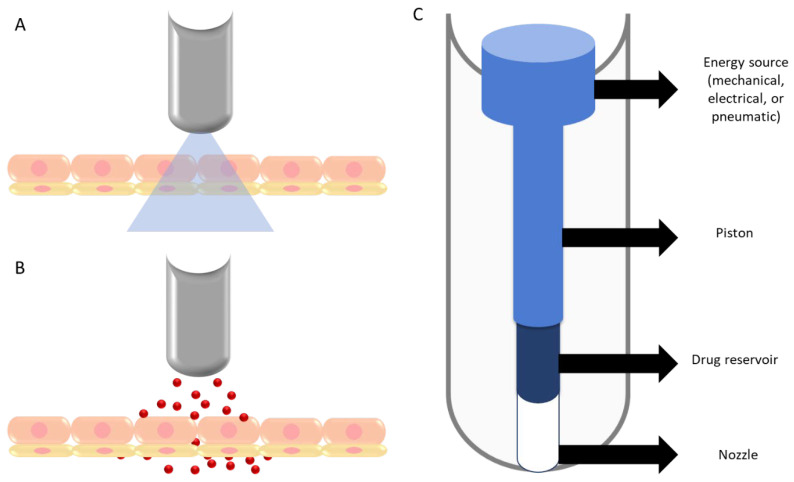
Graphical representations of (**A**) a liquid jet injector and (**B**) a powder Injector. (**C**) The internal anatomy of such devices. Figure Reprinted with permission from [278].

The liquid jet injector consists of a compartment (drug reservoir) which holds a drug formulation, a piston, a power source such as a spring or compressed gas, and an actuation mechanism consisting of a piezoelectric transducer which controls liquid delivery volume and injection velocity (Figure 7A) [275]. To deliver the drug formulation, the liquid within the compartment is compressed and pushed through a narrow orifice of 100 to 300 μm diameter [272]. By varying the jet velocity and orifice diameter, the jet injector can transport drug molecules intradermally, subcutaneously, or intramuscularly [14]. The jet syringe can be single- or multi-dose [39]. Multi-use nozzle jet injectors (MUNJIs) have been used for mass immunization against various diseases, such as measles, smallpox, cholera, hepatitis B, influenza, and polio [275]. Although MUNJIs allowed repeated injections of vaccines from the same nozzle and reservoir at a rate of >1000 immunizations per hour, its use was discontinued due to cross-contamination. Therefore, disposable-cartridge jet injectors, which separate disposable and reusable components, have been evolved to eliminate cross-contamination [275,276]. A series of Injex™ devices are currently marketed as liquid-based needle-free jet injectors with springs for the subcutaneous delivery of insulin. Injex30 and Injex150 differ in the volume of drug delivered to the skin: Injex30 delivers 0.05–0.3 mL insulin, whereas Injex150 delivers 0.8–15 mL insulin [277]. Moreover, liquid jet injectors can be used to deliver vaccines to dermal, subcutaneous, and muscular regions [278]. Nevertheless, the use of jet injectors has been limited due to variable reactions at the administration site, such as soreness, redness, and swelling [43,276].

The powder jet injectors are devices used to deliver vaccines or drugs, particularly water-sensitive drugs, in a dry powder or micro/nanoparticles at a speed of 600–900 m/s [275,277]. The powder jet injectors are also known as biolistic injectors, similar to the gene guns that are commonly used for DNA delivery (Figure 7B) [275]. These injectors have a basic design that include compressed gas, a drug compartment, and a nozzle that directs the flow of drug particles [279,280]. When using this device, the drug formulation, placed between two membranes, is pushed upon triggering the actuation mechanism and the membranes are ruptured, creating pressure along with the gas. The flow of gas carries the drug particles and delivers them through the skin [39,275]. The extent of penetration of drug particles through the skin varies based on the momentum of particles within the gas. This is because particles create micro-sized holes in the SC by virtue of their momentum. In addition, the physical properties of particles (size and density) play a role in determining the depth of penetration; some particles may deposit in the SC, while others may reach the viable epidermis [14,39,275]. The size of drug particles suitable for this technique was found to be between 10 and 20 μm, and for DNA vaccination was found to be between 0.5 and 3 μm. Additionally, particle densities of 1.08–18.2 g/cm^3^ have been used for powder injectors. It has been shown that increasing the size of particles decreased the depth of penetration. Furthermore, the typical range of pressure for powder jet injectors was estimated to be between 200 and 900 psi [275]. One of the major advantages of powder jet injectors is that the administration of drugs or vaccines in a solid state increases formulation stability and reduces the need for cold or freezing storage conditions, thus facilitating transportation and its associated costs [14]. The PowderJect™ injector has been used to successfully deliver testosterone, lidocaine hydrochloride, and macromolecules such as calcitonin and insulin [281]. Moreover, the use of powder injectors has been investigated for immunization against protein- and nucleotide-based antigens and downregulating allergic responses [275].

Despite their wide use, jet injector treatments have drawbacks which limit their commercial adaptation; jet injector devices are painful to operate, complex, and expensive. Reusable jet injector devices tend to be heavy and inconvenient, while the more convenient disposable variants have been criticized for being wasteful, raising environmental concerns about their sustainability.

#### 2.2.3. Mechanical Approaches

Microneedles (MNs) have received a lot of interest because of their painlessness and ease of usage for patients [282,283,284]. MNs were first proposed for drug delivery many decades ago but they did not become the target of extensive research until the mid-1990s, when microfabrication technology made them possible [8,285,286,287]. MN technology was developed to offer a delivery technique that was as reliable as hypodermic needles but without pain and other drawbacks [288].

MNs are microscopic projections that disrupt the top layer of the skin in a non-invasive manner, creating micron-sized channels ranging in height from 25 to 2000 µm that allow drugs to reach the epidermis or upper dermis directly [283,286,289]. MNs have facilitated the transdermal delivery of not only low-molecular weight drugs [290,291] but also hydrophilic molecules [292,293], peptides and proteins [294,295,296,297], cosmeceuticals [298,299,300], microparticles [301,302], NEs [303], vaccines [304], and nanoparticles [285,305].

MNs are classified into four categories based on their drug delivery methods: solid, coated, dissolving, and hollow MNs [11,291,297]. They can also be fabricated with a variety of materials, such as silicon, ceramics, glass, metal, sugars, and polymers, and have different lengths to accommodate different treatment sites and depths [287,306]. Needle length can be adjusted so that it penetrates the SC without reaching nerve ends [11,306]. Pain intensity and sensory perception were assessed using a visual analog scale (VAS) in a single-blind study involving 12 subjects comparing pain and sensation following the application of a 25G hypodermic needle and two MN arrays (36 needles, 180 and 280 m in length). The VAS pain scores revealed that the 180 and 280 m MNs caused significantly less discomfort than the hypodermic needle [307]. Many studies of MNs for transdermally delivering low- and high-molecular weight drugs have been conducted to date, employing diverse manufacturing methods and materials [11]. Tran et al. [308] described the development of a method for the pre-programmed release of a vaccine over a period of days and up to more than a month from a single dose using MNs made from poly(lactic-co-glycolic acid) with varied degradability kinetics. MNs containing a clinically available vaccine (Prevnar-13) generated immune responses in rats that were similar to those seen after numerous subcutaneous bolus injections and resulted in immune protection [308]. Dissolving MNs made entirely of polyvinylpyrrolidone with or without dissolution modifiers were recently categorized by Kathuria et al. [309]. Several grades of pharmaceutical cellulose, such as hydroxypropyl methylcellulose and methyl cellulose, have been studied as dissolution modifiers integrated into MNs [309]. Rates of dissolution differed depending on the pharmaceutical cellulose grades. Subsequently, dissolving MNs were classified as quick (45 min), moderately slow (2 to 2.5 h), slow (4 to 8 h), and extremely slow (>16 h), based on their dissolution period.

A more recent advancement in microneedle fabrication arose from the use of the various additive manufacturing technologies (collectively referred to as ‘3D printing’) as means to fabricate MNs. Three-dimensional printing is a layer-wise fabrication process in which each consequent layer is superimposed upon the previous layer via a material deposition nozzle [310]. Due to the micron-scale (and in some variants nano-scale), precision of 3D printing as a manufacturing method, MNs manufactured by 3D printing often cause less tissue damage and may be fabricated to have intricate hollow channels that are far more complex than what can be achieved via conventional methods, yielding greater control over release rates [311].

While the properties and the dimensional accuracy of 3D-printed MNs would largely depend on the particular 3D printing technology used [311], the differences, advantages, and limitations of the different types of 3D printing techniques are beyond the scope of this review. Nonetheless, various research efforts have demonstrated the capacity and robustness of 3D printing techniques to deliver a multitude of starkly different biologicals and small molecules via microneedle arrays. Pere et al. [312] reported the use of 3D-printed MNs to transdermally deliver insulin. Azizoglu et al. [239] designed a 3D-printed montelukast microneedle patch that can be printed directly on packaging material. Additionally, Economidou et al. [313] designed 3D-printed hollow microneedle arrays that were incorporated into a micro-doser electromechanical system for the personalised, transdermal delivery of drug solutions. Furthermore, Caudill et al. [314] demonstrated the feasibility of 3D MNs to transdermally deliver vaccines, citing that the increased skin contact time of the microneedle patch resulted in greater vaccine cargo retention and autoimmune response. Due to the high precision of 3D printing, giving the ability to design hollow MNs with channels of diameters in the nanometer scale, 3D-printed MNs are often designed to be coupled with dose–pump systems to better tailor the dosing and control the release rate of drugs delivered by electromagnetic pumps [311,312,313,315,316,317,318,319]. Derma roller was the first commercialized microneedle product. There are numerous microneedle products on the market that are approved mainly for cosmetic applications [320]. However, there are no microneedle products for drugs or vaccines. The lack of scaled-up GMP manufacture, regulatory hurdles, and lack of investment from pharmaceutical companies are the major challenges facing the microneedle market. LTS Lohmann now have the first GMP manufacturing license for microneedles, which raises prospects for a new direction and breakthrough in the field of TDD [321].

### 2.3. Integrating Chemical and Physical Technologies

Physical techniques have been exploited as a unique TDD platform for successful drug penetration in the treatment of a variety of disorders [10,14]. However, difficulties such as hydrophobic drug-loading capacity limitations, stability issues, and unpredictable drug-release rates, limit the use of physical methods. Drawing inspiration from the ways in which nanomedicine combined with physical approaches created new paths for disease therapy, the use of nanomedicine, in particular, can alleviate a number of issues associated with drugs, including poor solubility, poor stability, low bioavailability, and nonspecific distribution throughout the body [14,91,164]. Therefore, integrating physical with chemical technologies is a huge step forward from conventional TDDs, which are currently only viable for highly potent drugs. Once completely developed, this technology will have the potential to greatly extend the number of drugs that may be administered transdermally, which would be beneficial to both patients and industry.

Microparticles and nanoparticles are being used in a new generation of MNs to help achieve long-acting benefit after delivery into the body [309]. To provide targeted and long-acting intradermal distribution of amphotericin B for the treatment of cutaneous fungal infections, dissolving MNs loaded with micronized particles of amphotericin B were developed by the Donnelly research group [301]. Amphotericin B concentrations in plasma, kidneys, liver, and spleen were significantly lower in the MN group than in the intravenous group, according to pharmacokinetic and biodistribution studies. As a result, this approach addressed the systemic adverse effects of intravenous amphotericin B injections by localizing the drug for a week inside the skin [301].

The use of ASDs for TDD has extended beyond chemical methods to MNs. Solid dispersion-assisted MNs (SAMNs) are hybrid systems that incorporate ASD formulations with MNs to achieve greater transdermal penetration. SAMs formulations can be broadly divided into two categories: substrated SAMNs and matrix SAMNs. The primary difference between substrated SAMNs and matrix SAMNs is that substrated SAMNs are coated with the polymeric ASD, while matrix SAMNs are fabricated out of the polymeric ASD. Substrated SAMNs (also known as coated MNs) are simple systems. The MN array, made from a biologically inert material, is coated with a drug–polymer solution [227,322,323,324]. The coating technique is functionally identical to solvent-casting approaches to make solid dispersions, except that in this case the substrate used for solvent casting is the MN. Matrix SAMNs are formulations in which the drug–polymer ASD is processed into the shape of a MN array. This has been achieved via either casting hydrogel-forming polymeric solutions into MN-shaped molds, injection-molding of ASDs, or, more recently, via 3D printing drug-loaded MNs [311,316,317,325,326,327,328].

Donnelly RF et al. [329] described hydrogel-forming MN arrays made from “super swelling” polymeric compositions [329]. They fabricated a MN formulation with improved swelling properties using Gantrez S-97, PEG 10,000, and Na_2_CO_3_, as well as a lyophilized wafer-like drug reservoir. These MN–lyophilized wafer formulations were tough and effective in penetrating skin, swelling a lot, while remaining intact after removal [329]. The utilization of such devices in conjunction with lyophilized wafer-type drug reservoirs has made it possible to deliver high doses of non-potent drugs. In addition, the researchers used ovalbumin as a model for protein delivery, which suggested that this technique may be used in macromolecular drug delivery and vaccine delivery [329].

## 3. Challenges and Future Prospects

TDD is a non-invasive delivery approach that is generally regarded as being easy to administer even in more vulnerable age groups, such as paediatric and geriatric patients, whilst circumventing some bioavailability concerns that arise from oral drug delivery due to poor absorbability and metabolism concerns. The huge surface area and accessibility of the skin make it a convenient and patient-friendly drug delivery target. Elimination of first-pass metabolism, stable delivery, improved patient compliance, reduced systemic drug interactions, sustained drug release, and generally greater therapeutic efficacy are all key advantages of transdermal delivery.

Despite this impressive growth, there remain severe hurdles that restrict the application of TDD to a select few drugs. Only a handful of drugs which employ chemical TDD approaches have been successfully commercialized. It is, perhaps, the manufacturing complexity of chemical TDD systems that bottlenecks commercialization; chemical TDD systems such as niosomes and nanocrystal are not the final dosage forms and must be converted into a suitable dosage form (i.e., patches, creams, gels, etc.) before being used. This addition of excipients, which are required for various dosage forms, increases the cost and complexity of the manufacturing process and introduces several points of failure, such as particle size optimization and drug leakage concerns, which must be very tightly controlled so as to avoid lowering the efficacy of the dosage form. In addition, chemical methods have a major constraint when it comes to the delivery of hydrophilic macromolecule such as insulin and other proteins.

Born out of the inherent restrictions of chemical TDD methods, physical methods have evolved into promising systems for physical drug delivery via the skin. The majority of these physical approaches are still in clinical trials and are intended to administer a wide range of drugs, especially hydrophilic drugs and macromolecules. To date, over 1000 clinical trials investigating transdermal drug delivery are indexed in the United States National Library of Medicine (NLM; clinicaltrials.gov). Table 4, below, lists the current “top 10” clinical trials relating to transdermal drug delivery as generated by the NLM.

Although there already are a few products on the market that use physical approaches, there are still many issues to be addressed in the large-scale manufacture and product development of the various physical methods. However, there remain prospects for widespread adaptation in the future, which is spearheaded, as previously mentioned, by LTS—the first pharmaceutical manufacturer to obtain GMP approval for a MN production facility.

Another approach that is currently being investigated to improve TDD is the hybridization of chemical approaches with physical approaches, though the investigation is still in its early stages. Currently, the use of MNs in combination with nanomedicine for TDD is limited to proof-of-concept pre-clinical studies, since there has been only one report of a phase 1 clinical trial of human proinsulin peptides coupled to gold NPs delivered intradermally using MNs in type 1 diabetes [331]. It is anticipated that a combination of nanomedicine and MNs will increase rapidly in the future due to the major attributes of nanomedicine and their promising clinical outcomes, but further research is needed to fully utilize the therapeutic and diagnostic potentials of this smart combination [332]. Given the advances made in terms of research into innovative enhancing approaches, it is possible that we are still only exploring the commercial potential of the transdermal drug delivery market. There are still regulatory issues that need to be addressed, as evidenced by the limited approvals granted by the FDA during the last several years and the products’ final outcomes as well as those product ideas that never made it to market, whether for financial reasons or doubts about their safety and/or technological complexity.

## 4. Conclusions

TDD is currently gaining in popularity as a delivery system for a variety of diseases, owing to its merits, such as noninvasiveness and self-administration and allowing for consistent drug distribution at predetermined and controlled rates. As a result, TDD technology is becoming popular in the pharmaceutical industry. Nanoparticles, liposomes, nanocrystal, niosomes, and nanoemulsions, are just some of the chemical transdermal delivery platforms that have been utilized to deliver drugs to the skin. These delivery platforms, however, occasionally come across the stratum corneum, which hinders hydrophilic molecules and macromolecules from penetrating intact skin. Physical enhancement techniques were shown to boost drug delivery to the systemic circulation, allowing the administration of a wide range of drugs, especially those which are typically difficult to deliver using chemical penetration enhancement approaches, such as macromolecules. However, there are currently only a few products on the market that use this approach. Nonetheless, there are future prospects for the wider adaptation of TDD as a feasible method for system drug delivery. There are still, however, some obstacles that need to be addressed, including the complexity of applications when employing a combination of transdermal devices and other drug-loaded formulations. Furthermore, scaled-up GMP and regulatory control over manufacture are required for some new transdermal techniques such as microneedles.

## Figures and Tables

**Table 1:** List of transdermal chemical penetration enhancers with active ingredients and mechanisms of action.

CPEs	Drugs Used	Mechanism of Action
Dimethyl sulphoxide	Hydrocortisone [51] Testosterone [52] Naloxone [53]	Disrupt the lipid bilayer of the SC Denature the proteins of the SC Change the intercellular keratin conformation of the SC
Azone	Ketoprofen [54] Dimethyl fumarate [55] 5-Fluorouracil [56]	Disrupt the lipid bilayer of the SC
Pyrrolidone	Ketoprofen [54] Lidocaine hydrochloride [57] Bupranolol [58]	Change the intercellular keratin conformation of the SC Change the solubility properties of the SC
Fatty acids	Flurbiprofen [59] Propranolol [60] Theophylline [61]	Interact with the lipid bilayer and change its packing
Alcohols	Nortriptyline hydrochloride [62] Thymoquinone [63] Lidocaine [64]	Alter drug solubility in the SC Increase drug partitioning in the SC Extract the lipids of the SC
Urea	Indometacin [65] Venlafaxine hydrochloride [66] Metronidazole [67]	Disrupt the lipid bilayer of the SC Increase the hydration of the SC Start keratolytic activity
Terpenes	Zidovudine [68] Dimethyl fumarate [55] Imipramine hydrochloride [69]	Disrupt the lipid bilayer of the SC Increase drug partitioning in the SC
Surfactants	Lorazepam [70] Foscarnet [71] L-Ascorbic acid [72] Dimethyl fumarate [55]	Change the intercellular keratin conformation of the SC Change the solubility properties of the SC Solubilize the lipids of the SC Disrupt the lipids and proteins of the SC
Cosolvents	Diclofenac sodium [36]	Enhance drug penetration by reducing the rheological properties of penetration enhancer-loaded Carbopol™ gels

**Table 2:** Drugs loaded into NEs, the types of NEs, the method of NE preparation, drug class, and the transdermal delivery systems.

Drug	Type of NE	Method of Preparation	Drug Class	TDD
Ibuprofen	O/W	Spontaneous emulsification	NSAID	NE [134]
Aceclofenac	O/W	Spontaneous emulsification	NSAID	NE [135]
Meloxicam	O/W	Spontaneous emulsification	NSAID	NE [136] NE-loaded gel [137]
Celecoxib	O/W	Spontaneous emulsification	NSAID	NE [138,139]
Ketoprofen	W/O	Spontaneous emulsification	NSAID	NE [140] NE-loaded gel [141]
Indomethacin	O/W	Spontaneous emulsification	NSAID	NE [142,143] NE-loaded gel [144]
Piroxicam	O/W	Spontaneous emulsification	NSAID	NE-loaded gel [145]
Thiocolchicoside	W/O	Spontaneous emulsification	Muscle relaxant with anti-inflammatory and analgesic effects	NE [146]
Carvedilol	O/W	Spontaneous emulsification	Congestive heart failure	NE-loaded gel [147] NE-loaded film [148]
Olmesartan	O/W	Spontaneous emulsification	Antihypertensive	NE [149]
Nitrendipine	O/W	Spontaneous emulsification	Antihypertensive	NE-loaded gel [150]
Caffeine	W/O	Oil phase titration method	Anticancer drug	NE [151]
Ropinirole hydrochloride	W/O	Spontaneous emulsification	Parkinson’s disease	NE [152]
Inulin	W/O	Not mentioned	Model drug	NE [153]
Glycyrrhizin	W/O	Spontaneous emulsification	Gastric ulcer	NE [154]
Dutasteride	O/W	Spontaneous emulsification	Prostate cancer	NE-loaded patch [155]
Tamoxifen citrate	O/W	Spontaneous emulsification	Anticancer	NE [156]
Granisetron hydrochloride	O/W	Spontaneous emulsification	Antiemetic	NE [157]
Terbinafine and citral	O/W	Spontaneous emulsification	Model drugs	NE-loaded gel [158]
Glibenclamide	O/W	Not mentioned	Antidiabetic	NE-loaded gel [159]
Imipramine and doxepin	O/W	Not mentioned	Local anesthetics	NE [160]
Hydrocortisone	O/W	Spontaneous emulsification	Corticosteroid	NE [161]
Atorvastatin	O/W	Spontaneous emulsification	Lower cholesterol	NE [162]
Apixaban	O/W	Spontaneous emulsification	Anticoagulant	NE [163]

**Table 3:** Research efforts demonstrating the flexibility of nanocrystal incorporation into various dosage forms.

Drug	Dosage Form	Reference
Apremilast	Gel	[218]
Luliconazole	Hydrogel patch	[219]
Dexamethasone	Nanosuspension	[220]
Glabridin	Nanosuspension	[204,221]
Beclomethasone	Nanosuspension	[222]
Ibuprofen	Gel	[202]
Flurbiprofen	Gel	[223]
Methotrexate	Gel	[217]
Methotrexate	MNs	[216]
Curcumin	Adhesive film	[213]
Curcumin	Nanosuspension	[224]

**Table 4:** Recent clinical trials investigating transdermal delivery systems. ^1^ Full clinical trial information can be accessed via a trial code by searching for the trial code/reference [330]. Data presented in this table is publically available and open access via https://ClinicalTrials.gov/ (accessed on 15 April 2022).

Rank	Title	Conditions	Interventions	Trial Code ^1^
1	Gabapentin Versus Transdermal Fentanyl Matrix for Chronic Neuropathic Pain	Neuropathic pain| Spinal stenosis	Drug: transdermal fentanyl matrix, gabapentin	NCT01127100
2	Transdermal Basal Insulin Patch Study in Type 1 Diabetes	Type 1 diabetes	Other: PassPort(R) Transdermal Insulin Delivery System	NCT00519623
3	Disease-modifying Potential of Transdermal nicotine in Early Parkinson’s Disease	Parkinson’s disease	Drug: nicotine transdermal patch	NCT01560754
4	Effect of Transdermal Magnesium Chloride on Quality of Life in Patients with Fibromyalgia	Fibromyalgia|fibromyalgia syndrome	Other: Transdermal Magnesium Chloride	NCT01968772
5	Granisetron Transdermal Patch for Prophylaxis of Delayed CINV	Chemotherapy-induced nausea and vomiting (CINV)	Drug: Granisetron transdermal patch|Drug: Palonosetron|Drug: Aprepitant|Drug: Fosaprepitant|Drug: Dexamethasone	NCT04912271
6	Granisetron Transdermal Patch for Prophylaxis of Nausea and Vomiting in Patients Receiving Oral Anticancer Agents	Chemotherapy-induced nausea and vomiting (CINV)	Drug: Granisetron Transdermal Delivery System	NCT04472143
7	Totally Transdermal Sedation in the Weaning from Remifentanil Infusion	Respiratory insufficiency|ventilator weaning|analgesics, opioid	Drug: Fentanyl Transdermal System|Drug: Remifentanil	NCT04204967
8	Granisetron Transdermal Patch System for Prevention of CINV by CapeOX	Chemotherapy-induced nausea and vomiting	Drug: Granisetron Transdermal Patch System	NCT05325190
9	Opioid Titration With 12.5 ug/h Fentanyl Transdermal Patch vs Orally Morphine for Opioid-Naive Patients with Moderate Cancer Pain	Opioid, moderate cancer pain, transdermal fentanyl, 12.5 ug/h, opioid-naive	Drug: 2.5 ug/h transdermal fentanyl|Drug: Oral immediate-released morphine	NCT04533243
10	Comparison of Blood Pressure Measurements Between Transdermal Optical Imaging and Standard of Care	Blood pressure	Device: Transdermal Optical Imaging	NCT04539860

## Data Availability

Not applicable.
